# Mmm-derived lipid-associated membrane proteins activate IL-1β production through the NF-κB pathway via TLR2, MyD88, and IRAK4

**DOI:** 10.1038/s41598-017-04729-y

**Published:** 2017-06-28

**Authors:** Yang Wang, Qi Wang, Yuan Li, Ying Chen, Jiari Shao, Nwankpa Nick, Chunyan Li, Jiuqing Xin

**Affiliations:** 1 Key Laboratory of Fermentation Engineering (Ministry of Education), Hubei Provincial Cooperative Innovation Center of Industrial Fermentation, College of Bioengineering, Hubei University of Technology, Wuhan, China; 2 National Contagious Bovine Pleuropneumonia Reference Laboratory, Division of Bacterial Diseases, State Key Laboratory of Veterinary Biotechnology, Harbin Veterinary Research Institute, CAAS, Harbin, China; 3 College of Resources and Environmental, Northeast Agricultural University, Harbin, China; 4 College of Veterinary Medicine, Northeast Agricultural University, Harbin, China; 5 College of Animal Science and Technology, Jilin Agricultural University, Changchun, China; 6 African Union, Pan African Veterinary Vaccine Center, Debre Zeit, Ethiopia

## Abstract

*Mycoplasma mycoides* subsp.*mycoides* (Mmm) is a pathogen that causes pneumonia, otitis media, and arthritis in young calves. Its pathogenesis is attributed in part to excessive immune responses. Mmm-derived lipid-associated membrane proteins (LAMPs) are potent inducers of the host innate immune system; however, interactions between Mmm-derived LAMPs as pathogenic agents, toll-like receptors (TLRs), and the signaling pathways responsible for activating inflammation and nuclear factor (NF)-κB have not been fully elucidated. Here, we analyzed the expression kinetics of interleukin (IL)-1β in Mmm-derived LAMP-stimulated embryonic bovine lung (EBL) cells and found that Mmm-derived LAMPs induced IL-1β expression. Subcellular localization analysis revealed the nuclear translocation of the NF-κB p65 subunit after EBL cells were stimulated with Mmm-derived LAMPs. Furthermore, a specific inhibitor assay demonstrated that NF-κB is required for Mmm-derived LAMP-induced IL-1β expression. Additionally, overexpression of TLR2, myeloid differentiation primary response gene 88 (MyD88), and IL-1 receptor-associated kinase 4 (IRAK4) increased IL-1β expression during LAMP stimulation, and TLR2-neutralizing antibodies reduced IL-1β expression in EBL cells during LAMP stimulation. Furthermore, LAMPs inhibited IL-1β expression following transfection with dominant-negative MyD88 and IRAK4 variants. These results suggested that Mmm-derived LAMPs activate IL-1β production through the NF-κB pathway via TLR2, MyD88, and IRAK4.

## Introduction

Contagious bovine pleuropneumonia (CBPP) is caused by *Mycoplasma mycoides* subsp.*mycoides* (Mmm)^1^. Infection results in severe pathological changes in the lungs and respiratory tract, leading to significant mortality and morbidity and thus greatly reducing animal welfare and livestock production^2^. CBPP is the only bacterial disease included on List A of communicable animal diseases issued by the Office International Des Epizooties (OIE)^3^. China, where CBPP was eradicated through the use of the Ben-181 vaccine, has been officially certified as a CBPP-free country by OIE. However, the disease still exists and spreads in many African contries^4^. Although many efforts have been taken to explore its pathogenesis, the molecular pathways of pathology and immune inflammatory responses associated with Mmm infection remains largely unknown^5^.

Adhesion to the host cell is a prerequisite for the colonization and infection of the host^6^. In our previous work, we identified an adhesion protein in Mmm^7^. After adhesion to EBL cells, innate immunity is the first line of defense against Mycoplasma infection^8^. Mycoplasma have no cell wall; thus, the bacterial modulins such as lipopolysaccharide, lipoteichoic acid are less likely to be potent activators of monocytes and lymphocytes. Lipid-associated membrane proteins (LAMPs) are mixtures of bacterial lipoproteins that are expressed on the cell surface and constitute the main structures for interacting with host cells^9^. LAMPs were demonstrated to be biologically active, and they are the most potent initiators of inflammatory reactions during Mycoplasma infection^8^. Understanding the molecular mechanisms of host cell stimulated by Mycoplasma-derived LAMPs will contribute important informations about the molecular pathogenesis of Mycoplasma infection^5, 8–10^. He *et al*. reported that *Mycoplasma genitalium*-derived LAMPs can activate nuclear factor (NF)-κB through toll-like receptors(TLRs) 1,2, and 6 and cluster of differentiation (CD)14 via a myeloid differentiation primary response gene 88 (MyD88)-dependent pathway^10^. The transcription factor NF-κB is a central inflammatory mediator that is essential for the induction of a variety of inflammatory genes in response to various pathogens and inflammatory cytokines^11^. One such cytokine is IL-1β, a pyrogenic cytokine that is produced during the innate immune response following pathogenic invasion^12^. IL-1β regulation by the NF-κB signaling pathway plays an important role in shaping the inflammatory response against pathogens^8^. Previous reports indicated that after 2 μg/mL *Mycoplasma bovis*-derived LAMPs stimulation, the expression of IL-1β reached the peak at 12 h. In this study, we found that 1 μg/mL of Mmm-derived LAMPs could stimulate IL-1β expression to the peak levels at 6 h. Although the disease caused by Mmm and *M. bovis* exhibited similar clinical symptoms and pathological anatomy^4, 6–8^, the expression kinetics of IL-1β in Mmm-derived LAMPs-stimulated EBL cells were different from *M. bovis*-derived LAMPs-stimulated EBL cells.

Embryonic bovine lung (EBL) cells have proven specially useful for the study of cattle-related infectious disease processes, including those caused by *M. bovis* ^6^. In our previous study, we used EBL cells to identify an adhesion factor of *M. bovis* and found that *M. bovis*-derived LAMPs induced IL-1β expression through the NF-κB pathway via TLR2 and MyD88^6, 8^. Here, we used EBL cells to investigate the molecular mechanisms by which Mmm-derived LAMPs induce IL-1β expression and discovered that Mmm-derived LAMPs induce NF-κB activation through TLR2-, MyD88-, and IRAK4-dependent pathways. The present study provides a basis for understanding the molecular pathways of pathology and immune inflammatory responses associated with Mmm infection.

## Results

### Mmm-derived LAMPs induce IL-1β expression in EBL cells

**Figure 1:**
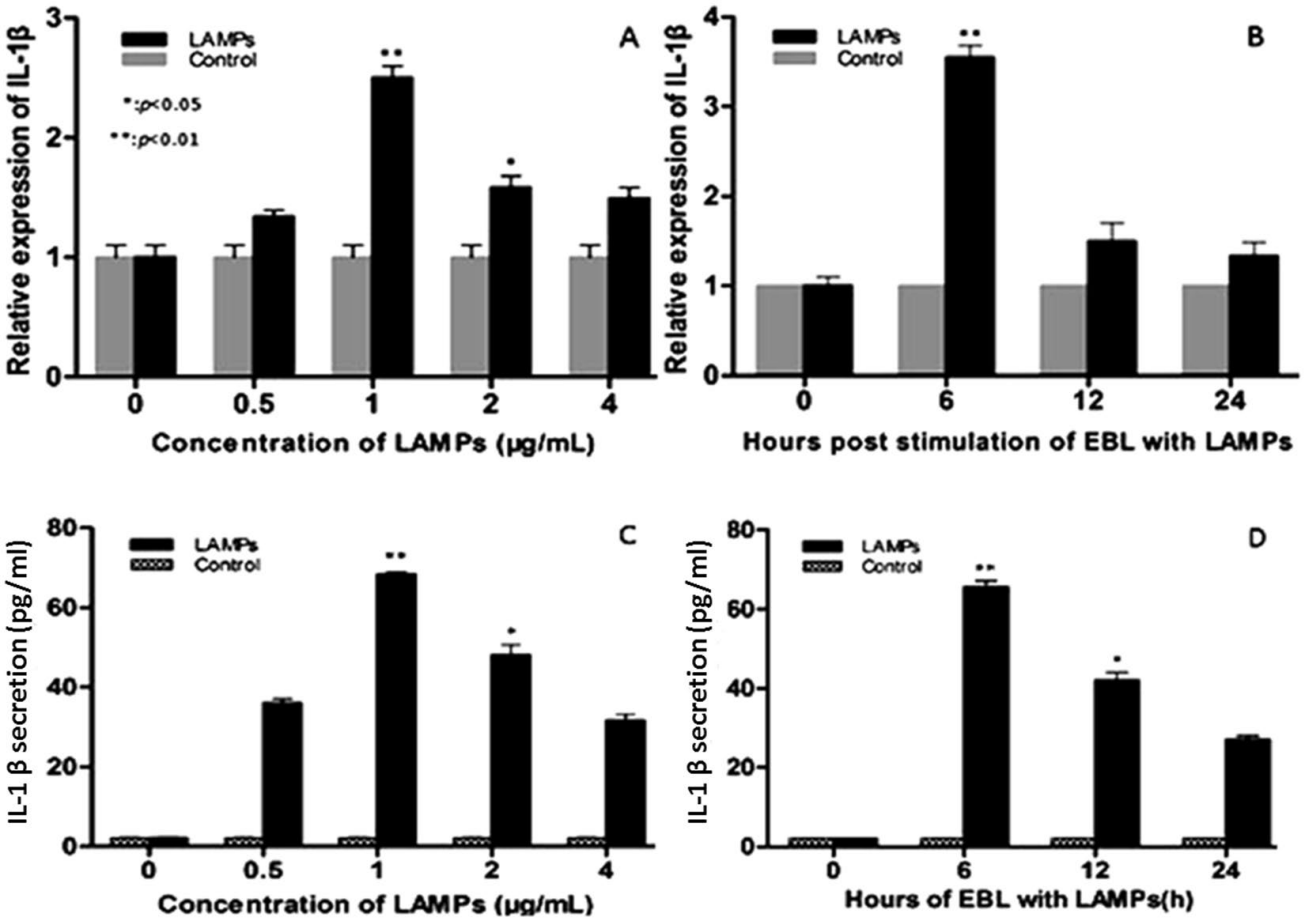
Mmm-derived LAMPs induce IL-1β expression. (**A** and **C**) EBL cells were cultured in serum-containing medium and then stimulated with the indicated concentrations of LAMPs for 6 h. Cells and supernatants were respectively harvested and analyzed by real-time PCR and ELISA. (**B** and **D**) EBL cells were stimulated with 1 μg/mL Mmm-derived LAMPs for 0, 6, 12, or 24 h (PBS was used as a negative control). The cells and supernatants were respectively harvested and analyzed by real-time PCR and ELISA. Data are presented as the mean and standard deviation of three assays. *p < 0.05 compared with PBS-treated cells. **p < 0.01 compared with PBS-treated cells.

We investigated the expression kinetics of IL-1β in Mmm-derived LAMP-infected EBL cells. Stimulation of EBL cells for 6 h with 1 μg/mL Mmm-derived LAMPs significantly upregulated IL-1β expression and secretion (Fig. 1).

### NF-κB is activated during LAMP stimulation

**Figure 2:**
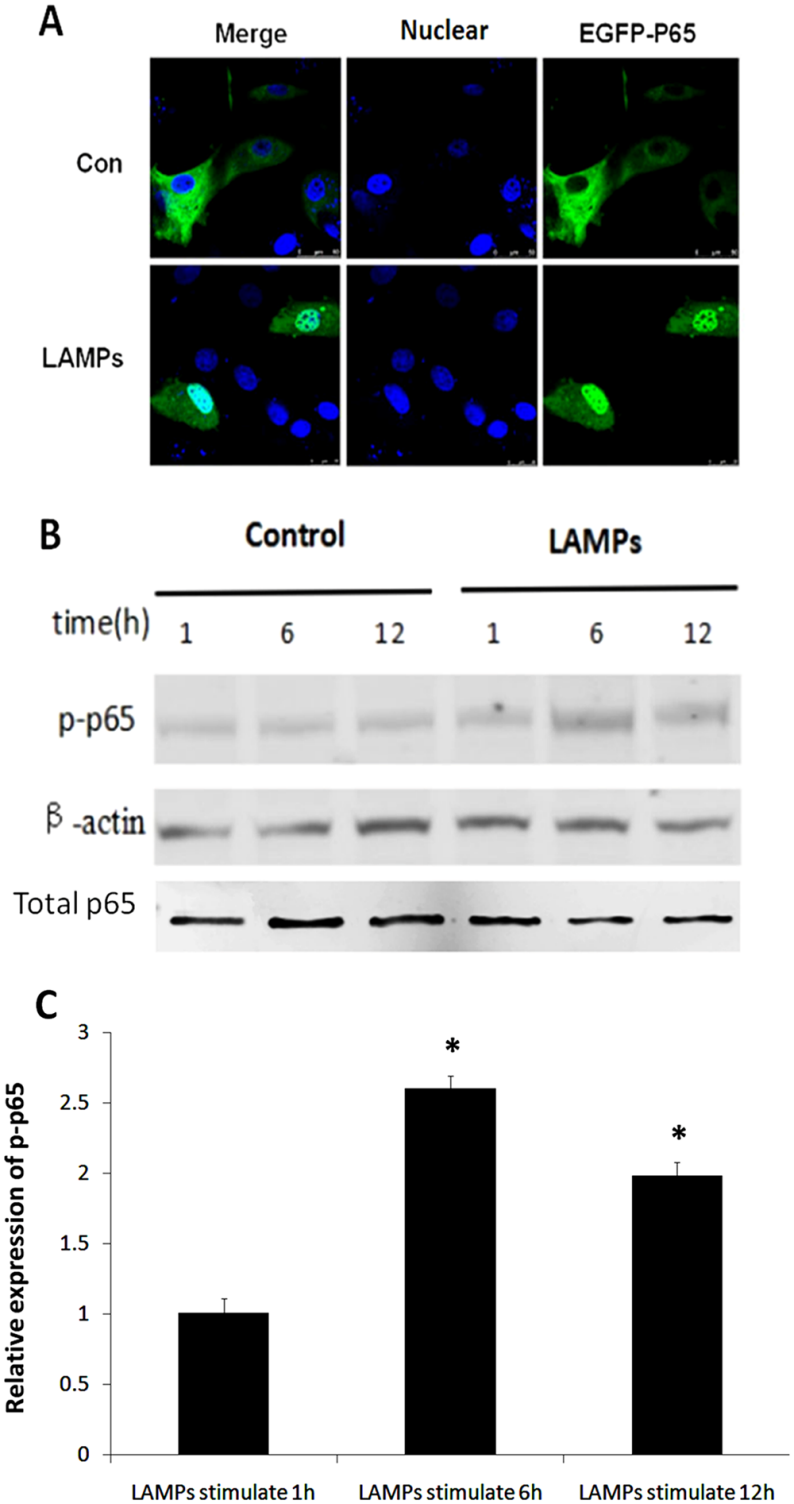
NF-κB is activated following LAMP stimulation. (**A**) EBL cells were transfected with an EGFP-p65 expression plasmid (pEGFP-p65) for 24 h and then stimulated with 1.0 μg/mL LAMPs for 6 h. Nuclei were counterstained with 1 μg/mL DAPI, and NF-κB nuclear translocation was observed using a laser-scanning confocal microscope. (**B**) EBL cells were stimulated for the indicated times with 1.0 μg/mL LAMPs, and Western blot analyses were performed with antibodies specific for bovine p65 and p-p65. β-Actin was used as a loading control. (**C**) Band densitometry results from the western blot shown in (**B**). β-Actin was used for normalization. *p < 0.05 (Student’s *t-*test).

NF-κB is an inducible transcription factor involved in pathogen- and cytokine-induced immune and inflammatory responses, as well as in cell proliferation and survival^8^. NF-κB activation is usually characterized by the nuclear translocation of NF-κB subunit p65^7^. To validate NF-κB nuclear translocation following Mmm-derived LAMP stimulation, EBL cells were transfected with EGFP-P65. As shown in Fig. 2A, p65 protein accumulated in the nucleus following LAMP stimulation, whereas it remained in the cytoplasm in the control group. We next utilized western blot analysis to show that phosphorylated p65 (p-p65) levels were increased in LAMP-stimulated cells. Total p65 levels were unchanged (Fig. 2B,C). These results indicated that Mmm-derived LAMPs induce NF-κB activation, as evidenced by p65 translocation.

### NF-κB mediates LAMP-induced IL-1β expression

**Figure 3:**
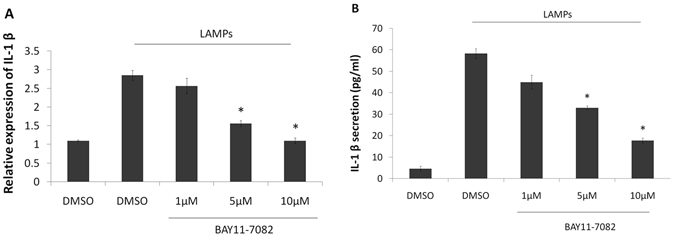
The NF-κB signaling pathway regulates Mmm-derived LAMP-induced IL-1β expression in EBL cells. EBL cells were stimulated or not with 1 μg/ml Mmm-derived LAMPs and then treated with an NF-κB inhibitor (1,5, or 10 μM) or DMSO in the absence of serum for 6 h. The cells and supernatants were respectively harvested and analyzed by real-time PCR and ELISA. Data are presented as the mean and standard deviation of three assays. *p < 0.05 compared with LAMP-stimulated cells.

To examine the role of the NF-κB signaling pathway in the regulation of IL-1β expression during Mmm-derived LAMP stimulation, EBL cells were stimulated with Mmm-derived LAMPs and subsequently treated with different doses of a specific NF-κB inhibitor (BAY11-7082; 1,5, and 10 μM). As shown in Fig. 3, BAY11-7082-treated cells exhibited a dose-dependent decrease in the upregulation of IL-1β expression following LAMP stimulation. This result indicated that NF-κB mediates the induction of IL-1β expression by Mmm-derived LAMPs in EBL cells.

### LAMP-induced IL-1β expression depends on TLR2

**Figure 4:**
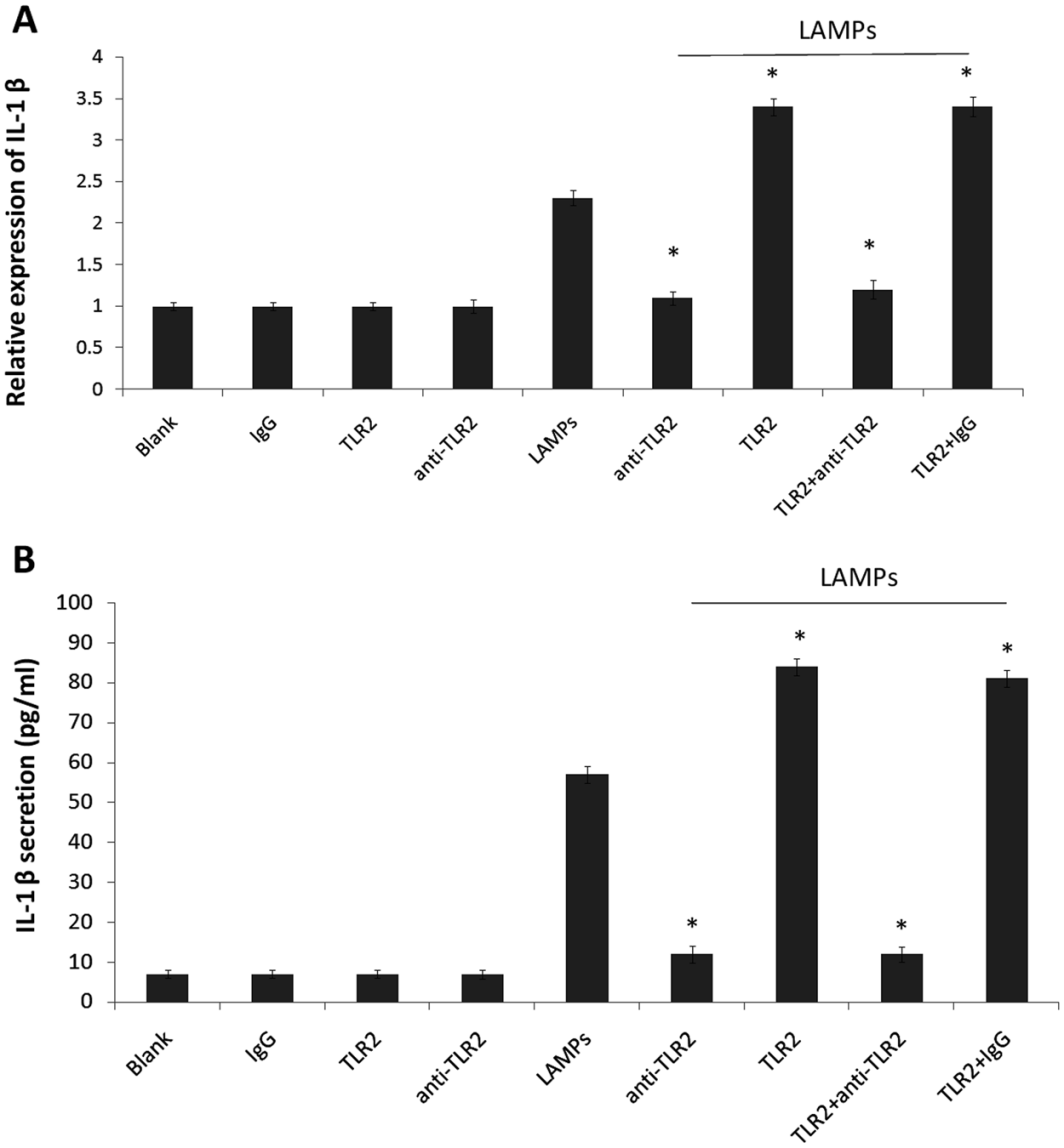
TLR2 is required for LAMP-induced IL-1β expression. EBL cells were transiently transfected with 0.2 μg/mL pHA-TLR2. After 24 h, the cells were pre-treated with 10 μg/mL human IgG or anti-TLR2 mAb (IgG) for 30 min and then stimulated with or without 1.0 μg/mL LAMPs. After 6 h, cells and supernatants were respectively harvested and analyzed by (**A**) real-time PCR and (**B**) ELISA. Data are presented as the mean and standard deviation of three assays. *p < 0.05 compared with the LAMP-stimulated group.

TLR2 is the initial molecule in a cascade of events leading to significant innate immune responses, the development of adaptive immunity to pathogens and protection from immune sequelae related to pathogen infection^13^. We previously cloned and sequenced bovine TLR2^8^. In this study, we observed that Mmm-derived LAMPs were able to induce IL-1β expression in EBL cells, and considered that TLR2 is usually involved in bacterial lipoprotein recognition. EBL cells were treated with an anti-TLR2 Ab (10 μg/mL) or isotype-matched human IgG (10 μg/mL) for 30 min, followed by stimulation with 1.0 μg/mL LAMPs for 6 h. LAMP-induced IL-1β expression and secretion decreased significantly following treatment with the anti-TLR2 Ab (Fig. 4).

To further evaluate the role of TLR2, we constructed a plasmid encoding TLR2 (pHA-TLR2) and transiently transfected it into EBL cells. After 24 h, the cells were pre-treated with 10 μg/mL isotype-matched human IgG or anti-TLR2 mAb (IgG) for 30 min and then stimulated with or without 1.0 μg/mL LAMPs. After 6 h, cells and supernatants were respectively harvested and analyzed by real-time PCR and ELISA. As shown in Fig. 4, TLR2 overexpression led to a marked increase in LAMP-induced IL-1β expression and secretion.

### LAMP-induced IL-1β expression depends on MyD88

**Figure 5:**
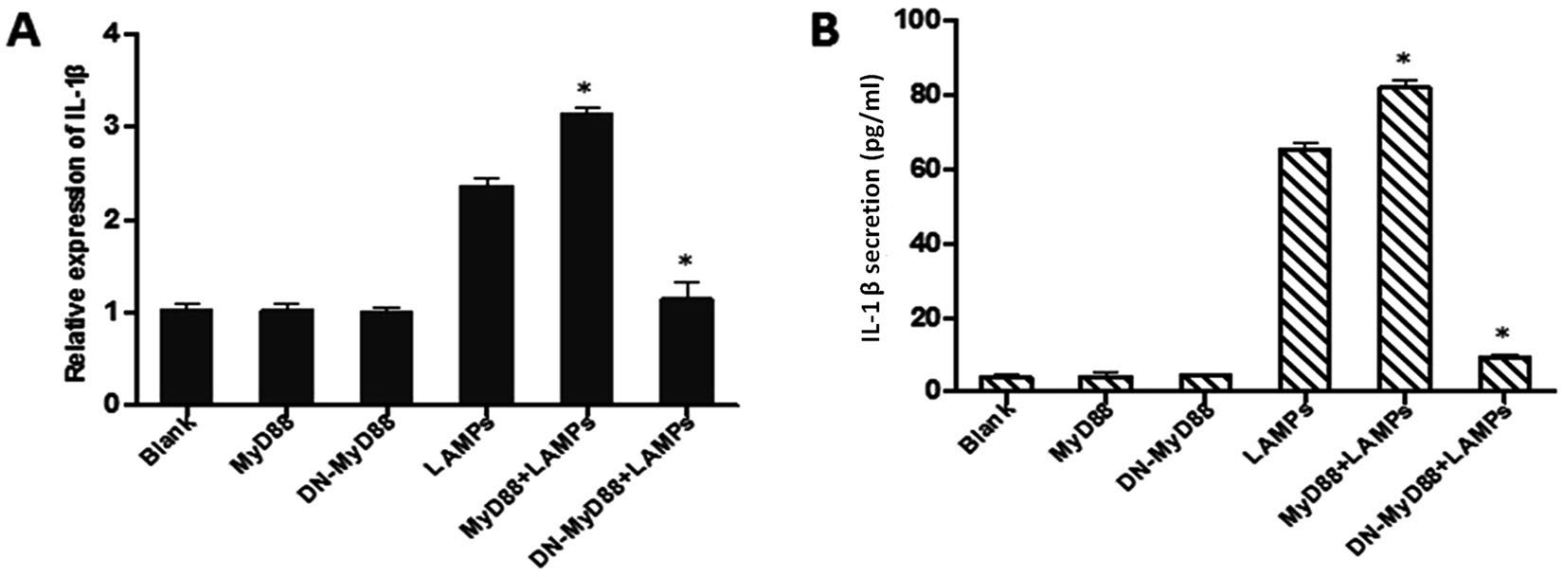
MyD88 is required for LAMP-induced IL-1β expression. EBL cells were transiently transfected with 0.2 μg/mL pHA-MyD88 or 0.2 μg/mL pHA-DN-MyD88. After 24 h, the cells were stimulated with or without 1.0 μg/mL LAMPs. After 6 h, cells and supernatants were respectively harvested and analyzed by (**A**) real-time PCR and (**B**) ELISA. Data are presented as the mean and standard deviation of three assays. *p < 0.05 compared with the LAMP-stimulated group.

Following ligand stimulation, TLR2 heterodimers generally initiate a MyD88-dependent intracellular pathway^12^. This pathway induces the nuclear translocation of NF-κB to modulate gene transcription and consequent inflammatory cytokine production^14^. Thus, we determined whether NF-κB induction occurs through MyD88 during Mmm-derived LAMP stimulation. EBL cells were transfected with MyD88 or DN-MyD88 and then incubated with 1.0 μg/mL LAMPs for 6 h. As shown in Fig. 5, MyD88-transfected cells exhibited significantly increased LAMP-induced IL-1β expression compared with control cells, while transfection with DN-MyD88 significantly decreased IL-1β expression compared with control.

### LAMP-induced IL-1β expression depends on IRAK4

**Figure 6:**
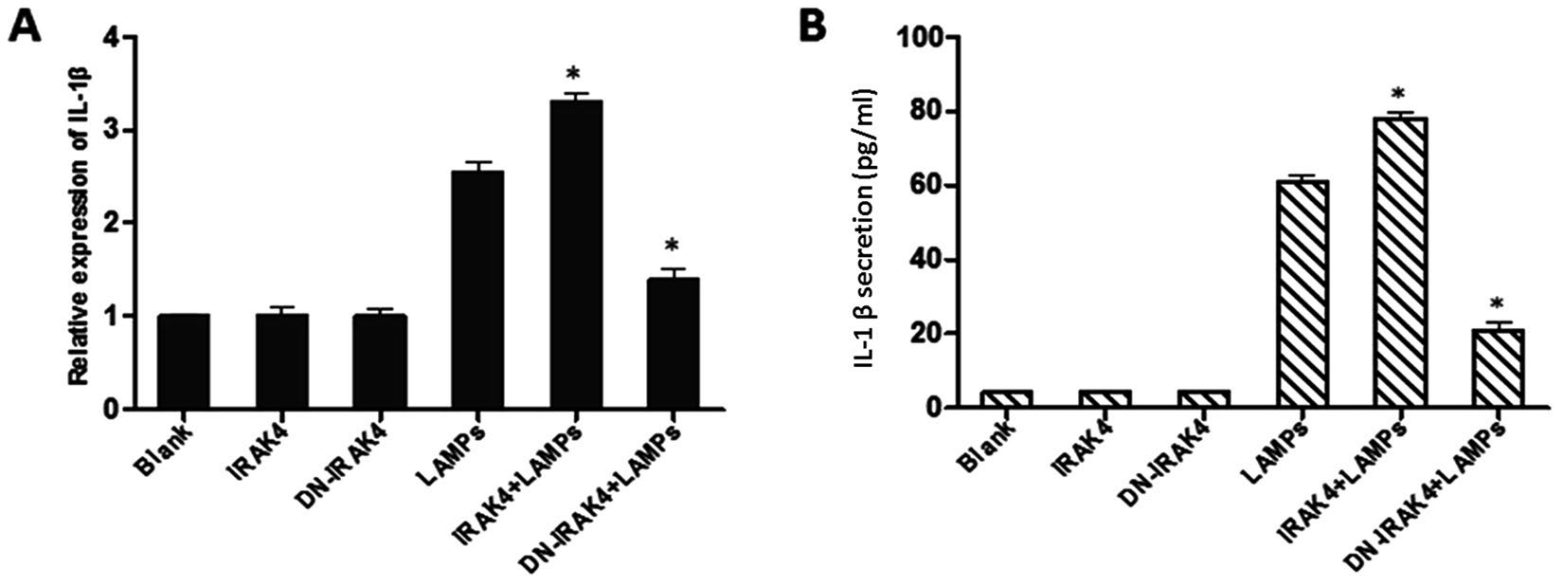
IRAK4 is required for LAMP-induced IL-1β expression. EBL cells were transiently transfected with 0.2 μg/mL pHA-IRAK4 or 0.2 μg/mL pHA-DN-IRAK4. After 24 h, the cells were stimulated with or without 1.0 μg/mL LAMPs. After 6 h, cells and supernatants were respectively harvested and analyzed by (**A**) real-time PCR and (**B**) ELISA. Data are presented as the mean and standard deviation of three assays. *p < 0.05 compared with the LAMP-stimulated group.

Upon ligand binding to TLR, the adaptor molecule MyD88 is recruited to the TLR complex as a dimer^12^. Then, MyD88 recruits interleukin-1 receptor (IL-1R) associated kinase 1 (IRAK1), IL-1R associated kinase 4 (IRAK4), and tumor necrosis factor receptor-associated factor 6 (TRAF6), resulting in the production of inflammatory cytokines, such as IL-1β^15^. To evaluate the role of IRAK4 in IL-1β expression, we transiently transfected EBL cells with a plasmid encoding IRAK4 (pHA-IRAK4) and then treated these cells with 1.0 μg/mL LAMPs for 6 h. As shown in Fig. 6, LAMP-induced IL-1β expression was markedly increased by IRAK4 overexpression but considerably decreased by overexpression of DN-IRAK4.

### Mmm-derived LAMPs activate IL-1β production through the NF-κB pathway via TLR2, MyD88, and IRAK4

**Figure 7:**
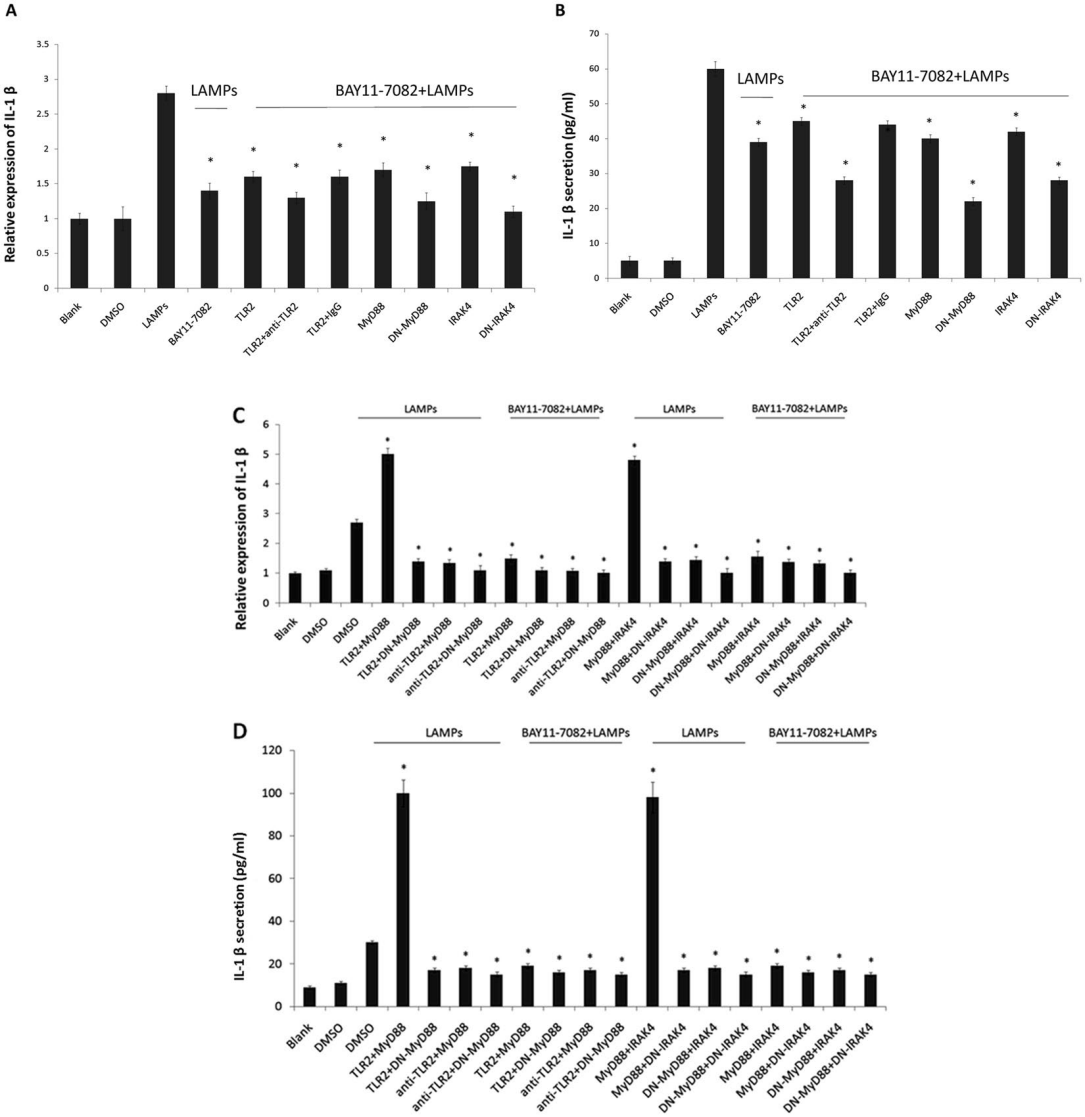
Mmm-derived LAMPs activate IL-1β production through the NF-κB pathway via TLR2, MyD88, and IRAK4. (**A** and **B**) EBL cells were transfected with 0.2 μg/mL pHA-TLR2, 0.2 μg/mL pHA-MyD88, 0.2 μg/mL pHA-DN-MyD88, 0.2 μg/mL pHA-IRAK4, or 0.2 μg/mL pHA-DN-IRAK4. After 24 h, the cells were incubated with 10 μg/mL of an anti-TLR2 mAb or an isotype-matched human IgG for 30 min, stimulated with 1.0 μg/mL LAMPs, and treated with the NF-κB inhibitor BAY11-7082 (10 μM) or DMSO (control) for 6 h. The cells and supernatants were then respectively harvested separately and analyzed by (**A**) real-time PCR and (**B**) ELISA. Data are presented as the mean and standard deviation of three assays. *p < 0.05 compared with DMSO-treated, LAMP-stimulated cells. (**C** and **D**) EBL cells were transiently cotransfected with the indicated constructs (0.2 μg/mL pHA-TLR2, pHA-MyD88, pHA-IRAK4, pHA-DN-MyD88, and pHA-DN-IRAK4). After 24 h, the cells were treated or not with 10 μg/mL anti-TLR2 IgG mAb for 30 min, stimulated with 1.0 μg/mL LAMPs, and treated with an NF-kB inhibitor (10 µM) or DMSO (vehicle control) in the absence of serum for 6 h. The cells and supernatants were then respectively harvested and analyzed by (**C**) real-time PCR and (**D**) ELISA. Data are presented as the mean and standard deviation of three assays. *p < 0.05 compared with DMSO-treated, LAMP-stimulated cells.

EBL cells were treated for 30 min with an anti-TLR2 IgG mAb (10 μg/mL) or an isotype-matched human IgG (10 μg/mL), transfected with the TLR2 plasmid, stimulated with Mmm-derived LAMPs, and finally treated with the NF-κB-specific inhibitor BAY11-7082. As shown in Fig. 7A and B, BAY11-7082-treated cells exhibited decreased upregulation of TLR2-dependent IL-1β expression after LAMP stimulation. TLR2 interacts with the MyD88 adaptor to elicit downstream signaling events^12^. MyD88 recruits IRAK4, which activates NF-κB and promotes the production of inflammatory cytokines, such as IL-1β^15^. Therefore, we assessed whether NF-κB activation by Mmm-derived LAMPs is dependent on MyD88 and IRAK4. EBL cells were transfected with 0.2 μg/mL of vectors expressing MyD88, DN-MyD88, IRAK4, or DN-IRAK4; stimulated with 1.0 μg/mL LAMPs; and treated with BAY11-7082 or DMSO (control). BAY11-7082-treated cells showed impaired MyD88- and IRAK4-dependent IL-1β induction following stimulation with Mmm-derived LAMPs (Fig. 7A,B).

Furthermore, EBL cells were cotransfected with 0.2 µg/mL pHA-TLR2, 0.2 µg/mL pHA-MyD88 or 0.2 µg/mL DN-MyD88. After 24 h, the cells were incubated with 10 µg/mL anti-TLR2 mAb or an isotype-matched human IgG (10 µg/mL) for 30 min, stimulated with 1.0 µg/mL LAMPs, and then treated with the NF-κB inhibitor BAY11-7082 (10 µM) or DMSO (vehicle control) for 6 h. Overexpression of both TLR2 and MyD88 significantly increased LAMP-dependent IL-1β expression compared with control (Fig. 7C,D). Pretreatment with the anti-TLR2 mAb almost completely inhibited the MyD88-dependent IL-1β induction following stimulation with LAMPs (Fig. 7C,D). DN-MyD88-overexpressing cells showed almost completely decreased TLR2-dependent IL-1β expression following stimulation with Mmm-derived LAMPs (Fig. 7C,D).

EBL cells were cotransfected with 0.2 µg/mL pHA-MyD88 or 0.2 µg/mL DN-MyD88 and 0.2 µg/mL pHA-IRAK4 or 0.2 µg/mL DN-IRAK4. After 24 h, the cells were stimulated with 1.0 µg/mL LAMPs and then treated with the NF-κB inhibitor BAY11-7082 (10 µM) or DMSO (vehicle control) for 6 h. Overexpression of both MyD88 and IRAK4 significantly increased LAMP-dependent IL-1β induction compared with control. Cotransfection with DN-MyD88 almost completely inhibited IL-1β induction following stimulation with LAMPs. DN-IRAK4-overexpressing cells showed almost completely decreased MyD88-dependent IL-1β expression following stimulation with Mmm-derived LAMPs (Fig. 7C,D).

## Discussion

Mmm enters and colonizes respiratory epithelial cells of cattle when they inhale aerosols in contaminated air^4, 16^. We previously determined that the P19 protein contributes to Mmm adherence to EBL cells^7^. Adhesion to epithelial cells is the first step required by pathogenic Mycoplasma to initiate an infection in human and animals^6, 7, 17^. After adhesion to EBL cells, innate immunity is the first line of defense against Mycoplasma infection^6^. LAMPs, which are abundant on the surface of Mycoplasma, may be the predominant proteins responsible for interacting with various components of the surrounding environment^8^. During mycoplasma infection, LAMPs are much more apparent than single lipoproteins^10^. LAMPs can activate the production of numerous cytokines in a variety of cells because of the biology activity of Mycoplasma^8, 18^. The biological effects of cytokines are needed for pathogen clearance and induction of effective adaptive immunity^18^. Studying host cytokine secretion induced by Mycoplasma LAMPs is key to understanding the complicated mechanisms of Mycoplasma infection^8^. The NF-κB signaling pathway is important for regulating cytokine production and has been widely studied in the context of activation by Mycoplasma LAMPs^8, 17^. For example, Mulongo *et al*.^17^ reported that monocyte NF-κB expression is activated after *M. bovis* infection, and You *et al*.^18^ reported that *M. genitalium*-derived LAMPs evoke IL-1β production in THP-1 cells through the NF-κB and mitogen-activated protein kinase signaling pathways. Notably, TLR2 has been implicated as a major response factor in host cells against Mycoplasma LAMPs^8, 10^. He *et al*.^10^ reported that *M. genitalium*-derived LAMPs activate NF-κB signaling in THP-1 cells through TLR1, TLR2, TLR6, and CD14 in a MyD88-dependent manner. We previously reported that *M. bovis*-derived LAMPs induce IL-1β expression in EBL cells via NF-κB signaling through TLR2 in a MyD88-dependent manner^8^. TLR2 utilizes the MyD88 adaptor, and MyD88 recruits IRAK4, which results in the activation of NF-κB and the production of inflammatory cytokines, such as IL-1β^14, 15^. In this study, we found that stimulation with Mmm-derived LAMPs resulted in IL-1β production through the NF-κB pathway via TLR2, MyD88, and IRAK4. Interestingly, although both Mmm- and *M. bovis*-derived LAMPs could induce IL-1β expression in EBL cells, the expression patterns of IL-1β were different. After 2 μg/mL *M. bovis*-derived LAMPs stimulation, the expression of IL-1β reached the peak at 12 h, whereas 1 μg/mL Mmm-derived LAMPs stimulation could induce the expression of IL-1β reached the peak at 6 h. This suggests that the activation components in Mmm- and *M. bovis*-derived LAMPs are different. LAMPs are mixtures of lipoproteins that are expressed on the cell surface and constitute the main structures for interacting with host cell^5^. In our previous study, we have sequenced the whole genomes of *Mycoplasma bovis* ^19^ and Mmm^4^. Thirty-nine lipoproteins and 47 lipoproteins were respectively identified in Mmm^4^ and *M. bovis* ^19^. TLR2 plays an indispensable role in lipoprotein-induced inflammatory responses^20^. The present study demonstrated that Mmm-derived LAMPs activate IL-1β production through the NF-κB pathway via TLR2, MyD88, and IRAK4. The potential interactions between identifed lipoproteins with TLR2 need further studies.

## Conclusion

In this study, we demonstrated that Mmm-derived LAMPs activate IL-1β production through the NF-κB pathway via TLR2, MyD88, and IRAK4. These results may increase our understanding of the complex mechanisms underlying the host immune response during Mmm infection.

## Materials and Methods

### *Mycoplasma* strain, LAMP preparation, cell line, and culture

*Mycoplasma* was cultured in modified pleuropneumonia-like organism (PPLO) medium supplemented with 20% inactivated horse serum (HyClone, Logan, WV, USA), 10% yeast extract, thallium acetate (0.125 mg/mL), and penicillin (200 IU/mL)^6, 7^. The origin and growth conditions of the EBL cells were described previously^6^, and LAMPs were prepared as described previously^8^. Briefly, Mmm was cultivated in PPLO medium until the beginning of the stationary phase, at which time the sample was pelleted by centrifugation for 10 min at 12,000 g. The pellets were washed with endotoxin-free phosphate-buffered saline (PBS), resuspended in 5 mL Tris-buffered saline [TBS; 50 mM Tris-Cl (pH 8.0) and 0.15 M NaCl] containing 1 mM EDTA (TBSE), solubilized with Triton X-114 at a final concentration of 2%, and incubated at 4 °C for 1 h. The lysate was incubated at 37 °C for 10 min for phase separation. After centrifugation at 10,000 × *g* for 20 min, the upper aqueous phase was discarded and replaced with the same volume of TBSE. The solution was then vortexed and incubated at 4 °C for 10 min, and the phase separation procedure was repeated twice. The final Triton X-114 phase was resuspended in TBSE to the original volume, a 2.5-foldvolume of ethanol was added to precipitate membrane components, and the solution was incubated at 20 °C overnight. After centrifugation, the pellet was resuspended in endotoxin-free PBS and then sonicated for 30 s. The protein concentration of the suspension was measured with Coomassie protein assay reagent (Pierce; Thermo Fisher Scientific, Waltham, MA, USA). The LAMP endotoxin concentration of the heat-inactivated mycoplasma was <0.04 endotoxin units/mL, as determined by the Limulus amebocyte lysate assay (Associates of Cape Cod, Inc., Falmouth, MA, USA). Both LAMP preparations were stored at −70 °C.

### Reagents and plasmids

The NF-κB inhibitor (BAY11-7082) was purchased from Sigma-Aldrich (St. Louis, MO, USA) and was dissolved in DMSO prior to use. Total RNA were extracted from the EBL cells with Trizol (Invitrogen) then quantified using the NanoDrop 1000 Spectrophotometer (Thermo Fisher Scientific Inc., USA). The quality of the RNA was checked by formaldehyde denaturing gel electrophoresis in 1.2% agarose gels, which showed dispersed bands (28S and 18S) without any obvious smearing patterns that would indicate degradation. 5 μg of total RNA were used for first strand cDNA synthesis by using Superscript II cDNA amplification System (Invitrogen) following manufacturer’s instructions. The bovine p65 gene was amplified from cDNA with the following primer pair: the forward primer 5′-AGACTCGAGCTATGGACGACCTCTTCCC-3′ and the reverse primer 5′-GGGGTACCTTAGGAGCTGATCTGACTCAG-3′; the bovine TLR2 gene was amplified form cDNA with the following primer pair: 5′-GGAATTCGGATGCCACGTGCTTTGTGGAC-3′ and the reverse primer pair: 5′-CCGCTCGAGCTAGGACCTTATTGCAGCTC; the bovine MyD88 gene was amplified from cDNA with the following primer pair: the forward primer 5′-GCGTCGACCATGGCTGAAGGAGTAC and the reverse primer 5′-GAAGATCTTCAGGGCATGGACAGGGC; the bovine IRAK4 gene was amplified from cDNA with the following primer pair: the forward primer 5′-GCGTCGACCATGAACAAACCCATAACAGC and the reverse primer 5′-GCAGATCTTTAAGAACCTGTCATTTCTTCTAGC-3′. Bovine P65 cDNA was subcloned into a vector containing enhanced green fluorescent protein (pEGFP; Invitrogen, Carlsbad, CA, USA) using the *Xho*I*/Kpn*I sites to generate the pEGFP-P65 construct. Bovine TLR2 cDNA was subcloned into the pCMV-HA vector (Invitrogen) using the *EcoR*I*/Xho*I sites to generate the pHA-TLR2expression construct. Bovine MyD88 and IRAK4 cDNAs were individually subcloned into the pCMV-HA vector (Invitrogen) using the *Sal*I*/Bgl*II sites to generate the pHA-MyD88 and pHA-IRAK4 expression constructs. Dominant-negative MyD88 (DN-MyD88) and dominant-negative IRAK4 (DN-IRAK4) were constructed according to the description provided by He *et al*.^10^.

### Antibodies and reagents

Neutralizing anti-human TLR2 mAb (ab45054) and isotype-control mAb [immunoglobulin G (IgG)] were used as blocking Abs and were purchased from Abcam (51AB; Shanghai, China). All other chemicals were obtained from commercial sources and were of analytical or reagent grade. Anti-p65, anti-p-p65 and anti-β-actin antibodies (1:1000) were purchased from Cell Signaling Technology (Danvers, MA, USA).

### RNA extraction and real-time quantitative analysis of IL-1β

Total cellular RNA was extracted from EBL cells using TRIzol reagent (Invitrogen), and genomic DNA was digested after RNA extraction. RNA was reverse transcribed into cDNA using reverse transcriptase (Roche, Basel, Switzerland), and cDNA was amplified using SYBR Green PCR assays (Roche). The primer sequences were as follows:IL-1β sense, 5′-CTAGCCCATGTGTGCTGAAG-3′; IL-1β antisense, 5′-CCTTTACTTGGCTCTTCACC-3′; GAPDH sense, 5′-ATCTCTGCACCTTCTGCCGA-3′; and GAPDH antisense, 5′-GCAGGAGGCATTGCTGACA-3′. Each cDNA amplification was performed in triplicate. PCR amplifications was performed using a Roche Light Cycle 480 Real-Time System. Thermal cycling conditions were as follows: 10 min at 95 °C, followed by 40 cycles of 10 s at 95 °C, 30 s at 56 °C, and 15 s at 72 °C. Gene expression was measured as a relative quantity as described previously^7^.

### Measurement of secreted IL-1β protein

IL-1β secretion into cell supernatants was measured using a commercial bovine IL-1β enzyme-linked immune sorbent assay (ELISA) reagent kit (Thermo Fisher Scientific) according to the manufacturer’s instructions.

### Western blot

EBL cells were seeded at 4 × 10^5^ cells/well in six-well tissue culture plates and incubated until they reached approximately 70% to 80% confluence. Transfections were performed using Lipofectamine 2000 reagent according to the manufacturer’s protocol. At 24 h post-transfection, the cells were washed twice with PBS, harvested, lysed, and boiled in sodium dodecyl sulfate (SDS) protein sample buffer [2% SDS, 10% glycerol, 60 mM Tris-HCl (pH 6.8), 0.001% bromophenol blue, and 0.33% β-mercaptoethanol]. The LAMP-stimulated cells were treated identically. Cell lysates were separated by 10% SDS-polyacrylamide gel electrophoresis, followed by electroblotting onto a nitrocellulose membrane. Western blots were performed with anti-p65, anti-p-p65 and anti-β-actin polyclonal antibodies and horseradish peroxidase-conjugated goat anti-rabbit IgG. Signals were visualized using a SuperSignal West Pico Luminol Kit (Pierce; Thermo Fisher Scientific). Bands were analyzed using the public ImageJ program (National Institutes of Health, Bethesda, MD, USA; available at http://rsb.info.nih.gov/ij).

### Confocal laser microscopy

EBL cells were grown to approximately 50% to 70% confluence on covers lips in 24-well plates. After transfection with 2.0 μg/well of p65-EGFP fusion expression construct, the cells were stimulated with 1.0 μg/mL Mmm-derived LAMPs for 6 h. Then, the cells were fixed with 4% paraformaldehyde for 10 min and permeabilized with 0.1% Triton X-100 for 10 min at room temperature. After three washes with PBS, the cells were incubated with 4′,6-diamidino-2-phenylindole-dihydrochloride (DAPI; Invitrogen) for 5 min at room temperature. After washing the slides with PBS, fluorescence images were acquired using a confocal laser scanning microscope (Leica TCS SP5; Leica, Mannheim, Germany).

### Transfection assay

Transient transfections were performed using Lipofectamine 2000 (Invitrogen). EBL cells were transiently transfected or cotransfected with the following constructs (each at 0.2 μg/mL): pHA-TLR2, pHA-MyD88, pHA-DN-MyD88, pHA-IRAK4, and pHA-DN-IRAK4. After a 24 h incubation, the cells were treated or not with 10 μg/mL anti-TLR2 mAb (IgG) for 30 min and then stimulated for 6 h with 1.0 μg/mL Mmm-derived LAMPs.

### Statistical analysis

Data are presented as the mean ± standard deviation. Statistical analysis was performed using Statistical Package Social Sciences (SPSS) version 17.0 (SPSS, Cary, NC, USA). A value of *p* < 0.05 was considered significant, and *p* < 0.01 was considered highly significant.
